# Supplementary material for: Entanglement propagation and dynamics in non-additive quantum systems
Source: Sci Rep. 2023 Jul 31;13:12388. doi: 10.1038/s41598-023-37984-3 (PMC10390585; doi:10.1038/s41598-023-37984-3)
Supplement: Supplementary file 1 — Supplementary Information. [file 41598_2023_37984_MOESM1_ESM.pdf]

Supplementary Material

1 Evolution of $\mu(t)$

Let us now derive the equation of motion for the effective mass $\mu(t)$. By tacking the time derivative of both sides of Eq. (3) of the main text

$$\begin{aligned}\dot{\mu} &= \frac{\lambda}{2N}\sum_k \mathcal{N}_k \dot{f}_k f_k^* + c.c. \\ \ddot{\mu} &= \frac{\lambda}{2N}\sum_n \mathcal{N}_k \left(|\dot{f}_n|^2 + \ddot{f}_n f_n^* + c.c.\right) \\ &= \frac{\lambda}{N}\sum_k \mathcal{N}_k |\dot{f}_k|^2 - \frac{\lambda}{N}\sum_k \mathcal{N}_k \left(\mu + \omega_k^2\right)|f_k|^2 \ ,\end{aligned} \tag{1}$$

from which, exploiting the conservation of energy and Eq. (3) of the main text, we can write

$$\ddot{\mu} = 2\epsilon + 2r\mu - 3\mu^2 - 2(\mu - r) + g(t) \tag{2}$$

where we introduced

$$g(t) = \frac{1}{2}\sum_k m_k |f_k(t)|^2, \tag{3}$$

and $m_k = 4\lambda\mathcal{N}_k(1-\omega_k^2)/N$. Let us notice that this equation, along with the equation of motion for the f_k, can be derived from the Hamiltonian (9) of the main text

$$H = \frac{P_\mu^2}{2} + V(\mu) - \sum_k \left(\frac{|p_k|^2}{2m_k} + \frac{m_k}{2}(\mu + \omega_k^2)|f_k|^2\right). \tag{4}$$

Now, $g(t) \sim \langle 1 - \omega_k^2 \rangle \sim O(N^{-\zeta})$ as long as $f_k = O(1)$, so that its contribution to the equations of motion of $\mu(t)$ is negligible. In this limit, we can thus set $f_k = 0$ in the above Hamiltonian and consider the single particle dynamics:

$$H = \frac{P_\mu^2}{2} + V(\mu). \tag{5}$$

Within the same approximation

$$\epsilon = \frac{\lambda}{2N}\sum_k \mathcal{N}_k |\dot{f}_k|^2 + \mu - r + \frac{1}{2}\mu^2 \tag{6}$$

up to $N^{-\zeta}$ correction. Let us notice that, the motion of this effective particle takes place within the bounded region of the potential, since the corresponding (conserved) energy $\mathcal{E} = \frac{\dot{\mu}^2}{2} + V(\mu)$ can only be negative. Indeed, by exploiting the Cauchy-Schwartz inequality, we find

$$\begin{aligned}\dot{\mu}^2 &= \left(\mathrm{Re}\left(\frac{\lambda}{N}\sum_k \mathcal{N}_k \dot{f}_k f_k^*\right)\right)^2 \leq \left|\frac{\lambda}{N}\sum_k \mathcal{N}_k \dot{f}_k f_k^*\right|^2 \\ &\leq \left(\frac{1}{N}\sum_k \mathcal{N}_k |f_k|^2\right)\left(\frac{1}{N}\sum_k \mathcal{N}_k |\dot{f}_k|^2\right).\end{aligned} \tag{7}$$

Now, putting together Eq. (6) and Eq. (3) of the main text and we find the constraint

$$\dot{\mu}^2 \leq 2\left(2\epsilon - 2(\mu - r) - \mu^2\right)(\mu - r) = -V(\mu), \tag{8}$$

from which the condition $\mathcal{E} < 0$ follows.

Let us now consider the modes $f_k = O(1)$. In this case the conserved energy becomes

$$\mathcal{E} = \frac{P_\mu^2}{2} + V(\mu) - \sum_k \frac{m_k}{2}\left(|\dot{f}_k|^2 + (\mu + \omega_k^2)|f_k|^2\right), \tag{9}$$

which differs from the single-particle energy for a quantity of order $N^{-\zeta}$. Now, if along the single-particle trajectory $\mu + \omega_k^2 > 0$ for every k, then the curve defined in the space of parameters f_k, $\dot{f}_k$, μ, $\dot{\mu}$ remains close to the $f_k = 0$ trajectory: in this case then, no resonance is possible. In particular, since $\mu(t) > r$, this ensures that no resonance actually occurs in the $r > 0$ case. The same reasoning leads to the the condition $r + \omega_k^2 > 0$ to prevent the resonance of modes with $k > 0$.

2 Derivation of the ground state properties

Let us now derive the expression for $\mu_{\rm gs}$ and $\epsilon_{\rm gs}$. Since each oscillator Φ_k is now in the ground state, we have

$$\begin{aligned}
\langle \Phi_k(0)\Phi_{k'}(0)^\dagger \rangle &= \frac{1}{2\sqrt{\omega_k^2+\mu_{\rm gs}}}\;\delta_{k'k},\\
\langle \Pi_k(0)\Pi_{k'}(0)^\dagger \rangle &= \frac{1}{2}\sqrt{\omega_k^2+\mu_{\rm gs}}\;\delta_{k'k},\\
\langle \Phi_k(0)\Pi_{k'}(0) \rangle &= \frac{i}{2}\;\delta_{k'k}
\end{aligned} \tag{10}$$

from which, exploiting Eqs. (22) of the Methods, we find

$$f_k(0) = \left(\omega_k^2+\mu_{\rm gs}\right)^{-1/4}, \qquad \dot f_k(0) = i\left(\omega_k^2+\mu_{\rm gs}\right)^{1/4}, \tag{11}$$

valid up to an immaterial phase factor. Since $\mu_{\rm gs}$ is now a positive constant, the solution of Eqs. (23) of the Methods is given by

$$f_k(t) = \left(\omega_k^2+\mu_{\rm gs}\right)^{-1/4} e^{i\sqrt{\omega_k^2+\mu_{\rm gs}}\,t}. \tag{12}$$

Finally, from Eq. (3),

$$\mu_{\rm gs} = r + \frac{\lambda}{2N}\sum_k \frac{1}{\sqrt{\omega_k^2+\mu_{\rm gs}}}. \tag{13}$$

In our case we can replace $\sqrt{\omega_k^2+\mu_{\rm gs}}$ with $\sqrt{1+\mu_{\rm gs}}$, up to $O(N^{-\zeta})$ corrections, obtaining:

$$\mu_{\rm gs} = r + \frac{1}{2}\frac{\lambda}{\sqrt{1+\mu_{\rm gs}}}. \tag{14}$$

This always has a unique solution. Since we are implicitly assuming $\mu_{\rm gs} > 0$, in order to have an oscillatory behavior for the $k=0$ mode, we have to require $r > -\lambda/2$. For $r < -\lambda/2$ the $k=0$ mode acquires a non-zero occupation number, signalling the emergence of a finite magnetization. The fact that the system undergoes a phase transition even in one dimension is not surprising, since the Mermin-Wanger theorem no longer holds in presence of long-range interactions. The corresponding energy per particle is, up to $O(N^{-\zeta})$ correction, given by

$$\epsilon_{\rm gs} = \frac{\lambda}{2N}\sum_k \frac{1}{\sqrt{\omega_k^2+\mu_{\rm gs}}}\left(2\omega_k^2+\mu_{\rm gs}\right) + \frac{1}{2}\mu_{\rm gs}^2 = \frac{1}{2}\frac{\lambda}{\sqrt{1+\mu_{\rm gs}}}\left(2+\mu_{\rm gs}\right) + \frac{1}{2}\mu_{\rm gs}^2. \tag{15}$$

This allows for a simple physical interpretation: indeed $\epsilon_{\rm gs}$ is such that $V'(\mu_{\rm gs}) = 0$, so that the ground state corresponds to the stable equilibrium for the fictitious particle in the potential $V(\mu)$.

3 Von Neumann entropy for a single resonance

We now apply the procedure exposed in Methods. In our case, from Eq. (10) of the Methods we have

$$\gamma_{\rm red} = \frac{1}{2}\begin{pmatrix} Q(t) & R(t) \\ R(t) & P(t)\end{pmatrix}, \tag{16}$$

where $Q(t)$,$P(t)$,$R(t)$ are ℓ by ℓ matrices defined as

$$\begin{aligned}
Q(t) &= |f_\pi(t)|^2\mathbb{I}_\ell + \frac{\ell}{N}|\dot f_\pi(t)|^2\mathbb{P},\\
P(t) &= |\dot f_0(t)|^2\mathbb{I}_\ell + \frac{\ell}{N}|\dot f_\pi(t)|^2\mathbb{P},\\
R(t) &= \mathrm{Re}\left(f_\pi(t)\dot f_\pi^*(t)\mathbb{I}_\ell + \frac{\ell}{N}f_0(t)\dot f_0^*(t)\mathbb{P}\right),
\end{aligned} \tag{17}$$

with $\mathbb{P}_{j,k} = \frac{1}{\ell},\ \forall\ j,k = 1,\dots\ell$. Since $[P,R]=0$ and $[Q,R]=0$ we find

$$-\left(J\gamma_{\rm red}\right)^2 = \frac{1}{4}\begin{pmatrix} PQ-R^2 & 0 \\ 0 & PQ-R^2\end{pmatrix}. \tag{18}$$

Using the fact that $\mathrm{Im}(f_k(t)\dot{f}_k^*(t)) = 1$ and $\mathbb{P}^2 = \mathbb{P}$ we have

$$PQ - R^2 = \mathbb{I} + \ell\Delta(t)\mathbb{P} + \ell^2 N^{-2}\,\mathbb{P}, \tag{19}$$

with $\Delta(t)$ of Eq. (12) of the main text. For a finite interval, the last term on the r.h.s. of Eq. (19) is negligible while the second one may become $O(1)$ on a timescale $t_q \sim \ln(N)$. Then all the eigenvalues of $-(J\gamma_{\mathrm{red}})^2$ are $\frac{1}{4}$ but two which are

$$\frac{1}{4} + \frac{\ell}{4}\Delta(t). \tag{20}$$

The symplectic spectrum is finally given by

$$\begin{aligned} \sigma_1 = \sigma_2 &= \frac{1}{2}\sqrt{1 + \ell\Delta(t)}, \\ \sigma_n &= \frac{1}{2} \quad \forall\ n = 3, \ldots 2\ell. \end{aligned} \tag{21}$$

Substituting in Eq. (29) of the Methods, we notice that only the first two eigenvalues, coming from the resonant mode, do actually contribute to $S(t)$, and we recover the expression (11) in the Main text.
